# S148. DIFFERENTIAL NEURAL REWARD MECHANISMS IN TREATMENT RESPONSIVE AND TREATMENT RESISTANT SCHIZOPHRENIA

**DOI:** 10.1093/schbul/sby018.935

**Published:** 2018-04-01

**Authors:** Lucy Vanes, Elias Mouchlianitis, Tracy Collier, Bruno Averbeck, Sukhi Shergill

**Affiliations:** 1 Institute of Psychiatry Psychology and Neuroscience, King’s College London; 2 National Institute of Mental Health; 3 Cognition Schizophrenia and Imaging (CSI) Lab

## Abstract

**Background:**

The significant proportion of schizophrenia patients refractory to treatment targeting the dopamine system suggests that more than one mechanism may cause psychotic symptoms. Reinforcement learning tasks have frequently been employed in schizophrenia to assess dopaminergic functioning and reward processing, but studies have not directly compared groups of treatment-refractory and non-refractory patients.

**Methods:**

In the current functional magnetic resonance imaging study 21 patients with treatment resistant schizophrenia (TRS), 21 patients with non-treatment resistant schizophrenia (NTR), and 24 healthy controls (HC) performed a probabilistic reinforcement learning task, utilising emotionally valenced face stimuli which elicit a social bias toward happy faces. Behavior was characterized with a reinforcement learning model. Trial-wise reward prediction error (RPE) signaling and the differential impact of emotional bias on these reward signals were compared between groups.

**Results:**

Patients showed impaired reinforcement learning relative to controls, while all groups demonstrated an emotional bias favouring selection of the happy faces. The pattern of RPE signaling was similar in HC and TRS groups, whereas NTR patients showed significant attenuation of RPE-related activation. The TRS patients differed from the NTR patients in the relationship between emotional bias and subcortical RPE signal during negative feedback.

**Discussion:**

TRS can be dissociated from NTR on the basis of a different neural mechanism underlying their symptoms. The data support the hypothesis that a favourable response to antipsychotic treatment may be contingent on dopaminergic dysfunction, characterized by aberrant RPE signaling, whereas treatment resistance may be characterized by an abnormality in distinct cognitive mechanisms interacting with this response.

